# The association between self-esteem and happiness differs in relationally mobile vs. stable interpersonal contexts

**DOI:** 10.3389/fpsyg.2014.01113

**Published:** 2014-10-09

**Authors:** Kosuke Sato, Masaki Yuki

**Affiliations:** ^1^Office for Students with Disabilities, Center for Student Counseling, Nagoya UniversityNagoya, Japan; ^2^Department of Behavioral Science, Graduate School of Letters, Hokkaido UniversitySapporo, Japan; ^3^Center for Experimental Research in Social Sciences, Hokkaido UniversitySapporo, Japan

**Keywords:** socio-ecology, relational mobility, happiness, self-esteem, culture

## Abstract

Does a change in the nature of surrounding social context affect the strength of association between self-esteem and happiness? This paper aims to answer this question from a socio-ecological perspective, focusing on the role of relational mobility. Recent research has shown that this association is stronger in societies that are higher in relational mobility, where there is a greater freedom of choice in interpersonal relationships and group memberships. In this study, we tested if this hypothesis could be applied to situational differences within the same physical setting. Using a quasi-experimental design, we tested if the association between self-esteem and happiness was stronger for first-year students at a Japanese university who had just entered the college and thus were in a relatively higher mobility context, than the second-year students at the same university whose relationships tended to be more stable and long-standing. The results showed, as predicted, that the association between self-esteem and happiness was stronger for the first-year students than for the second-year students. Implications for the theory and research on social change are discussed.

## INTRODUCTION

Happiness has long been a topic of attention, even back in ancient times. The nature of happiness has thus been a major topic of discussion and study in philosophy as well as the social sciences, such as psychology, sociology, and economics (e.g., Veenhoven,1999; Kahneman et al., 2006; Diener and Biswas-Diener, 2008; Inglehart et al., 2008; Lyubomirsky, 2008; Deaton, 2013). In psychology, there is an extensive amount of research into happiness, particularly focusing on its predictors. Among the wide number of factors that have been found to predict happiness, such as socioeconomic status, income inequality, and relational satisfaction, self-esteem is perhaps the one factor that has attracted the greatest attention from psychologists (James, 1890/1983; Rosenberg, 1965; Mecca et al., 1989; Kernis, 2006; Mruk, 2006). An abundance of empirical research conducted worldwide has revealed that self-esteem is a universal and crucial factor that is related to the level of happiness amongst people (Taylor and Brown, 1988; Baumeister et al., 2003; Cheng and Furnham, 2003; Brown, 2010) and thus generally, self-esteem has been found to be positively associated with happiness (e.g., Baumeister et al., 2003).

However, research also found that the magnitude of the association between self-esteem and happiness could be affected by a variety of factors. For instance, cross-cultural studies have found that this association varies between cultures (Diener and Diener, 1995; Kwan et al., 1997; Uchida et al., 2008). Hence, self-esteem tends to be more strongly associated with happiness in Western cultures particularly in North America than in East Asian cultures (Diener and Diener, 1995; Kwan et al., 1997; Uchida et al., 2008). Other research indicates that there can be significant cross-regional differences within one nation (Yuki et al., 2013, Study 2). However, whether the magnitudes of association between self-esteem and happiness have cross-situational variation remains unknown. The aim of the present study is to answer this question from a scoio-ecological perspective (Oishi and Graham, 2010; Yuki and Schug, 2012).

Given the variation described above an obvious question to ask is why do these differences exist? Cultural psychologists have typically explained the observed differences in terms of distinctions in shared meaning systems between these cultural regions, such as cultural self-construals (Markus and Kitayama, 1991, 2010). Namely, self-esteem strongly affects happiness in the culture of the “independent self,” which is prevalent in North American countries, since this culture emphasizes the importance of values attached to individuals. On the contrary, self-esteem does not affect happiness as much in East Asian countries where interdependent self-construal, which emphasizes the importance of interpersonal connections than personal values, is prevalent. However, an evident problem inherent in this kind of “explanation” is its tautological nature; the “explaining” (the importance of individual values) and the “explained” (the degree to which individual values are associated with happiness) are almost synonymous.

### A SOCIO-ECOLOGICAL APPROACH AND RELATIONAL MOBILITY

To address this problem, Yuki et al. (2013) have proposed an alternative account from a socio-ecological perspective. In general, the socio-ecological approach assumes that and analyzes how psychological and behavioral tendencies of individuals and characteristics of the social environment surrounding them influence each other (see Oishi and Graham, 2010, for an extensive review of this approach). This approach has recently been adopted for explaining various cross-cultural and cross-societal differences in behavioral and psychological tendencies (Nisbett and Cohen, 1996; Uskul et al., 2008; Oishi, 2010; Oishi and Schimmack, 2010; Gelfand et al., 2011).

Among a number of socio-ecological factors that has been proposed to date to explain various cultural differences, one that has recently attracted many researchers’ attention is relational mobility (Yuki and Schug, 2012). Relational mobility refers to the degree to which there is an availability of options in a given society or social context regarding interpersonal relationships, such as opportunities to acquire new, maintain current, and sever old relationships (Schug et al., 2010). There are abundant opportunities to meet strangers, and greater freedom of choice in selecting and changing whom to interact with, in high relational mobility societies, such as North America. In low relational mobility societies, such as East Asia, on the contrary, relationships, and group memberships tend to be stable and exclusive, and thus it is hard for individuals to replace them even when one wishes to do so. Researchers have found that relational mobility tends to be higher in North American than in East Asian societies, and have shown that this factor successfully explained various cross-cultural differences such as in the levels of general trust (Yuki et al., 2007), self-enhancement (Falk et al., 2009), similarity between friends (Schug et al., 2009), self-disclosure (Schug et al., 2010), shame proneness (Sznycer et al., 2012), and sensitivity to social rejection (Sato et al., in press).

### RELATIONAL MOBILITY AND DETERMINANTS OF HAPPINESS

Yuki et al. (2013) have proposed that the level of relational mobility in a given social environment should affect the magnitude of association between self-esteem and happiness, in that higher relational mobility should make this association stronger. Why should this be the case? The primary reasons are (1) an individual’s generic social value is critical for the interpersonal successes in high relational mobility contexts, and (2) self-esteem is a subjective indicator of a generic social value of the self. As discussed, in high relational mobility societies, people have many chances to meet strangers, who are sometimes socially attractive, and have opportunities to be associated with them. Although this type of society or social context might look attractive on surface, there is a drawback. That is, given the limitation in the number of social relations that an individual can properly maintain there is inevitably high interpersonal competitiveness to acquire from a relatively small number of desirable or beneficial partners, consequently not everyone can achieve the relationships they desire. The critically important point here is whether one can succeed or not in this context, depends on how attractive an individual is in the eyes of many others, who are both current and potential relational partners (otherwise, you might end up with someone else stealing your best friend or partner, as sung about in the melancholy lyrics of the Tennessee Waltz). And, what’s important, self-esteem is a *sociometer,* that is a subjective indicator of the generic social value of the self (Leary et al., 1995; Leary and Baumeister, 2000). Thus, self-esteem, or the perception that one is sufficiently attractive in the given social environment, should be associated with happiness particularly strongly in high relational mobility societies, because this value is more likely to lead to social success.

On the contrary, self-esteem should not be associated with happiness as strongly in low relational mobility societies because, as discussed, achievement and replacement of interpersonal relationships tends to be difficult, and thus whether an individual has high or low generic social value does not as significantly affect their chance for social success. Rather, the quality of current relationships, which are likely to continue into the future, should be more influential in determining happiness for those in low relational mobility context.

A series of studies by Yuki et al. (2013) found results consistent with this reasoning. First, the association between self-esteem and happiness was stronger among Americans than among Japanese, while the reversed pattern was true for the association between perceived relational quality and happiness, replicating previous research (Uchida et al., 2008). Importantly, these cultural differences were statistically explained by the difference in perceived relational mobility of participants’ immediate social environment (Study 1). Study 2 tested whether the magnitude of association between self-esteem and happiness would differ between local regions in the same country (i.e., Japan), varying in their relative degrees of relational mobility. Results showed that this association was stronger in areas where people more frequently changed their jobs than in those where job mobility was lower (Study 2). Study 3 experimentally manipulated participants’ perceived relational mobility using a priming technique, and tested if this manipulation would affect the degree of association between self-esteem and happiness. Supporting the prediction, it was found that this association was stronger when participants were asked to recall the situations when they had talked with strangers than when they had talked with their family (Study 3). There are, however, some weaknesses inherent in these studies. First, both Studies 1 and 2 showed that, as predicted, relational mobility of local and regional ecology moderated the strength of association between self-esteem and happiness, but there are a number of other possible confounding factors between the countries and regions, including language, economy, climate, etc. Second, although Study 3 used a priming technique and successfully indicated the predicted causal relationship between relational mobility and the association between self-esteem and happiness, the manipulation changed *perception* of relational mobility, not the actual levels of relational mobility that existed in the environment. Therefore, we conducted a new study, which sought to control for confounding factors as far as possible, employing a quasi-experimental method, which examined two similar groups of people who were facing two similar circumstances that crucially differed in the associated levels of relational mobility.

### PRESENT STUDY

In the present study, we aimed to test whether a change in the degree of relational mobility in the surrounding social context would affect the magnitude of association between individuals’ self-esteem and happiness. More specifically, using a quasi-experimental method, we observed if the strength of this association among Japanese students who belong to the same university might change between the time when they have just entered college and there are abundant chances to meet and select new friends (high relational mobility), and when they have entered their second year and relationships tend to be more stable and exclusive (low relational mobility). Although research has shown Japan to be a typical low relational mobility society, when compared with the United States (e.g., Schug et al., 2009, 2010; Yuki et al., 2013), there are a varieties of circumstances and places when and where relational mobility is particularly higher than average (Yamagishi et al., 2012; Yuki et al., 2013). The college situation right after entrance (i.e., the few months after April for the first-year students) can be one such typical situation.

This study is intended to address the limitation of the previous studies by Yuki et al. (2013) as first, it could exclude the historical or cultural confounding factors of specific regions (e.g., the United States vs. Japan or urban vs. rural). Thus, an important strength of the current study is that we could adopt a “just minimal difference” paradigm (Uskul et al., 2008) that allows us to keep constant as many potentially confounding variables as possible in order to test the hypothesis. For our comparison groups, we used different academic years from a sample of students from the same university. These students share the same general nature of living (i.e., all students live in the same city and its suburb and about the half of them live quite near to the university), the same academic stage, and similar daily lives and backgrounds (i.e., family income). Thus, we could control not only personal but also many surrounding socio-ecological characteristics with the exception of relative levels of relational mobility. The second advantage of our present approach was that it allowed us to test the hypothesis without attempting to induce some change in cognition or perception of the social environment but rather relied on a “real” change of social contexts.

In the present study, we specifically predicted that: (1) relational mobility should be higher among the first-year university students than among the second-years and (2) the magnitude of association between self-esteem and happiness should be stronger among the first-year than the second-year students. To the best of our knowledge, the present study is the first study to investigate the moderation effect of relational mobility on the association between self-esteem and happiness by comparing cross-contextual differences in relational mobility using a quasi-experimental design.

## MATERIALS AND METHODS

### PARTICIPANTS

Five hundred and thirty seven Japanese university students [344 first-years (251 male, 93 female), 193 second-years (110 male, 83 female)] participated in this study. The mean average age for the first-years was 18.47 (*SD*_age_ = 0.70) and 19.50 for the second-years (*SD*_age_ = 0.67).

### PROCEDURE AND MATERIALS

We conducted the survey at the end of April and the beginning of May, which is about a month after a school year starts in Japan. Given the generally low relationally mobile nature of interpersonal relationships among Japanese university students (e.g., Schug et al., 2010; Yuki et al., 2013), the first-year students at this time of the year are supposed to be experiencing one of the rarely relationally mobile contexts of their student lives.

Participants who volunteered to take the survey completed the questionnaire packet in a class setting. We administered two sets of questions in order to confirm our assumption that the first-year and second-year students resided in social environments that differed in their relative degrees of relational mobility. First, we asked participants about their perception of relational mobility in their immediate social environment using the relational mobility scale (Yuki et al., 2007). Sample items include: “They (i.e., people in my immediate society) have many chances to get to know other people” and “They can choose who they interact with.” Second, we asked the participants to report the number of new friends and acquaintances that they had made in the past month in order to provide an indirect self report measure of personal rather than perceived societal relational mobility (Schug et al., 2010). Finally, we asked participants’ about their happiness and self-esteem (in that order) employing the 5-item Satisfaction with Life Scale (Diener et al., 1985; Rosenberg’s,1965) 10-item scale, respectively. We chose these particular scales as they are the most frequently used and well-validated scales available to assess happiness (e.g., Diener and Diener, 1995) and self-esteem (e.g., Schmitt and Allik, 2005). Sample items from the satisfaction with life scale are, “In most ways my life is close to my ideal” and “I am satisfied with my life” and for the Rosenberg self-esteem scale, examples are “I feel that I am a person of worth, at least on an equal plane with others” and “I feel that I have a number of good qualities.” All measurements were made on 6-point scales ranging from 1 (strongly disagree) to 6 (strongly agree), with the exception of the question concerning the number of new acquaintances made.

## RESULTS

**Table 1** shows the scale means, standard deviations, and reliability coefficients for each scale. First, we calculated the reliability of items for each scale. All the scales were highly reliable: Rosenberg’s self-esteem scale (α = 0.84), satisfaction with life scale (α = 0.78), and relational mobility scale (α = 0.81). Thus, we calculated the average of each scales to generate an overall scale score.

**Table 1:** Means and standard deviations of, and intercorrelations among, variables by first vs. second-year students.

	*n*	*M*	*SD*	1	2	3	4
**First-years**
**1** Relational mobility	344	4.03	0.58	–			
**2** Self-esteem	344	3.51	0.73	0.14*	–		
**3** Satisfaction with life	344	3.75	0.87	0.12*	0.53***	–	
**4** # of new friends and acquaintances	336	40.03	29.57	0.14**	0.14**	0.15**	–
**Second-years**
**1** Relational mobility	193	3.88	0.55	–			
**2** Self-esteem	193	3.45	0.76	0.12^†^	–		
**3** Satisfaction with life	193	3.84	0.86	0.15*	0.38***	–	
**4** # of new friends and acquaintances	189	15.21	23.16	0.14^†^	- 0.02	0.23**	–

^†^p < 0.10; *p < 0.05; **p < 0.01; ***p < 0.001. Correlational coefficients between the number of new friends and acquaintances and other variables are Spearman’s r.

Second, we checked if the first-year and second-year students were, as expected, facing situations varying in the degree of relational mobility. As predicted, perceived relational mobility was higher among the first-years (*M* = 4.03, *SD* = 0.58) than among the second-years (*M* = 3.88, *SD* = 0.56), *t*(535) = 2.86, *p* = 0.004, *d* = 0.26. Moreover, the first-years self reported that they had obtained a greater number of new friends and acquaintances in the past month (*M* = 40.03, *SD* = 29.57) than the second-years (*M* = 15.21, *SD* = 23.16), *t*(523) = 10.64, *p* < 0.0001, *d* = 0.91 (**Table 1**)^1^^,^^2^ . These results confirmed our assumption that our first-year students were indeed in a social context higher in relational mobility than the second-year students.

**FIGURE 1:**
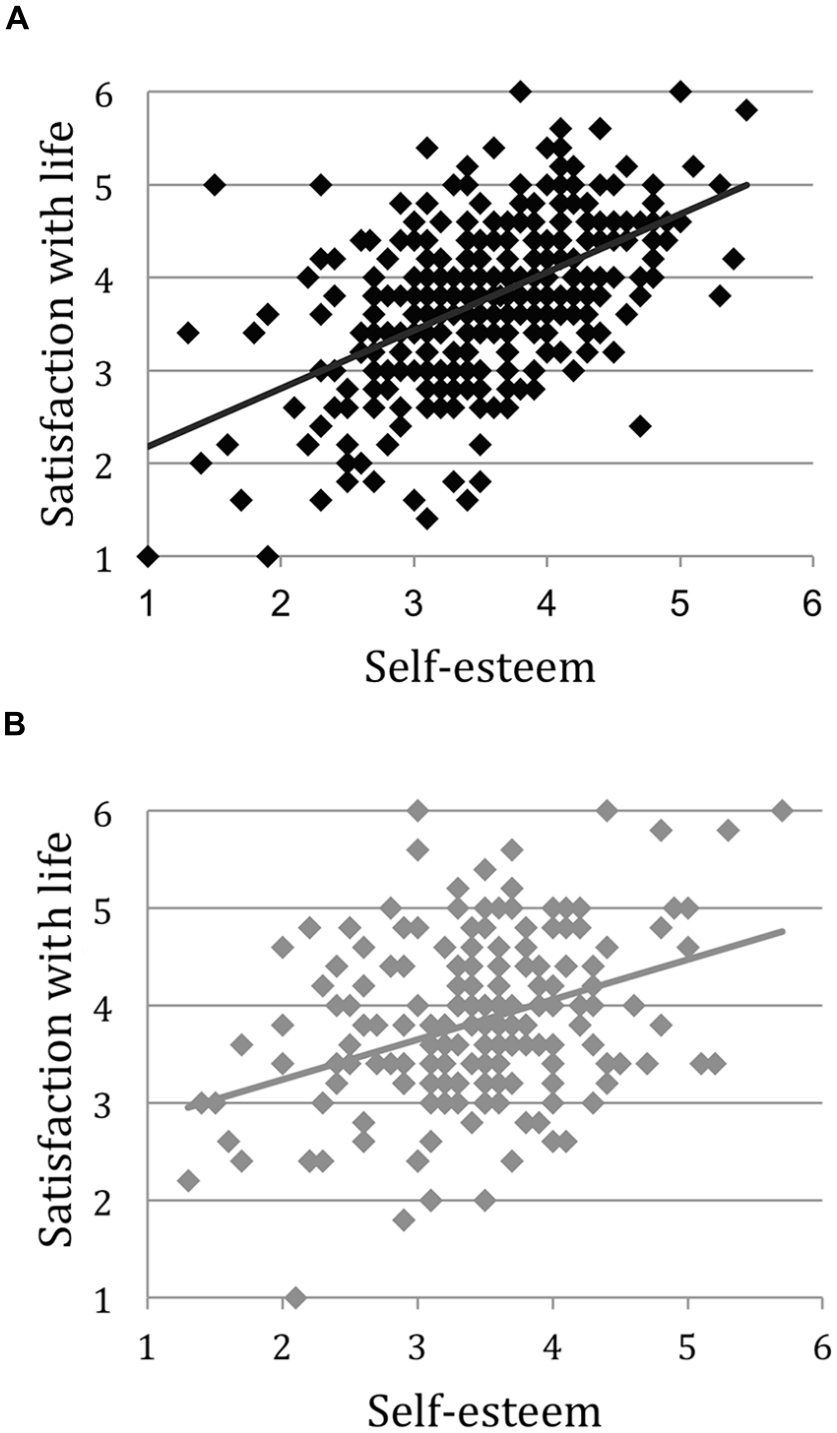
**Relationship between satisfaction with life and self-esteem for **(A)** the first-years, **(B)** the second-years**.

Next, as the main analysis, we compared the strengths of correlations between self-esteem and happiness between first- and second-years. As can be seen in **Table 2**, the correlation was stronger for first-year students (β = 0.53, *p* < 0.001) than for second-year students (β = 0.38, *p* < 0.001). This observation was confirmed by a multiple regression analysis where self-esteem, school year (dummy coded as first-year student = 1, second-year student = 0), and a self-esteem × school year interaction effect were entered as the independent variables, with happiness as the dependent variable. The results showed, in addition to the main effect of self-esteem, there was also a significant interaction effect between self-esteem and school year (β = 0.11, *p* = 0.026), with the strength of association between self-esteem and happiness stronger for first-year students than second-year students (**Figure 1**), thus supporting our prediction (**Table 2**: Fisher’s *z* regarding the difference in the strength of association = 2.10, *p* = 0.036). Importantly, the mean levels of self-esteem and happiness, as well as their variances, did not differ between the first-year and the second-year students [self-esteem: *t*(535) = 0.78, happiness: *t*(535) = -1.17, *p*s > 0.44, *d*s < 0.10; **Table 1**]. This rules out a possible alternative explanation that our results might have been due to the insufficient variability of self-esteem and/or happiness among second-year students.

**Table 2:** Regression analysis for satisfaction with life.

Variable	All		First-years		Second-years	
	*b*	β	*b*	β	*b*	β
Self-esteem	0.63***	0.54	0.63***	0.53	0.43***	0.38
School year (0 = second-years, 1 = first-years)	-0.12^†^	-0.07			–	
Self-esteem × School year	0.20*	0.11			–	
*R^2^*	0.24***	0.28***	0.14***

^†^p < 0.10; *p < 0.05; ***p < 0.001.

## DISCUSSION

In this study, we examined the role of relational mobility, a social-contextual factor pertaining to the degrees of freedom in selecting one’s relational partners, in affecting the determinants of levels of happiness among people. A study employing a quasi-experimental design showed that, first, the first-year college students who had just entered the university, as predicted, perceived that they were in a social context that was higher in relational mobility than the second-year students who had spent more than a year in the college. Moreover, also as predicted, the association between self-esteem and happiness was greater for the first-year students than the second-year students.

The significance of the current findings are twofold: first, they make a compelling case for Yuki et al.’s (2013) hypothesis that higher relational mobility in a social context generates a stronger association between self-esteem and happiness among people who reside there. Although Yuki et al. (2013, Studies 1 and 2) successfully demonstrated that this association was stronger in countries and regions (within a country) that are higher in relational mobility, factors other than relational mobility, which also differed between the relevant countries and regions, might have also played a role. The present study overcame this limitation, by comparing students at the same university, who were facing different contexts in their college life, specifically whether they were at the very beginning or with one year already completed. Adopting the “just minimal difference approach” (Uskul et al., 2008) generally makes it possible for researchers to rule out potential confounding variables that may exist between two different cultural regions, such as differences in language, economy, and climate, by keeping as many unrelated variables constant as possible across groups, making it more plausible to infer causality. Coupled with the findings from previous studies, this study therefore provides strong evidence that Yuki et al.’s (2013) hypothesis that higher relational mobility in a social ecology makes the association between self-esteem and happiness stronger among the people who reside there, a parsimonious explanation for why this association has been found to differ between countries (Kwan et al., 1997; Uchida et al., 2008; Yuki et al., 2013, Study 1), regions (Yuki et al., 2013, Study 2), and now between circumstances^3^.

Second, this is the first study to show the contextual effect of relational mobility on the degree of association between self-esteem and happiness. An implication from this is that the rapid change in relational mobility in many societies that have been traditionally characterized as low in relational mobility, due to “globalization” of the economy, information, human resources, and so on (e.g., Shirakawa, 2011, Chart 2; Yamagishi, 2011), may provide greater opportunities and freedom for people to meet strangers and to select and replace who to interact with more easily and thus increase relational mobility. For instance, Japanese society is experiencing increased divorce and remarriage (Japanese Ministry of Health, Labor, and Welfare Statistics and Information Department, 2014) as well as job turnover and wishing rates (Japanese Ministry of Health, Labor, and Welfare, 2006; Japanese Ministry of Internal Affairs and Communications, 2013), affording people with greater freedom to select whom to associate with. Therefore, it is possible that the strength of association between self-esteem and happiness, which has been found to be relatively weak among East Asians as compared to North Americans (e.g., Kwan et al., 1997; Uchida et al., 2008), may gradually be becoming stronger. Our socio-ecological approach thus offers a crucial perspective for predicting some of the psychological consequences of our rapidly changing world.

### LIMITATION AND FUTURE DIRECTIONS

We note, however, that some limitations of this research deserve to be mentioned. First, although we did our best to keep as many potentially confounding factors constant as possible, in comparing the first-year students who had just entered college and the second-year students who had spent a year at the same school, there might still be other variables that differ between them other than their relative level of relational mobility, such as age and the level of academic knowledge. Thus, future studies may want to employ a standard experimental method, randomly assigning participants to high vs. low relational mobility circumstances, and testing if the magnitude of association between self-esteem and happiness might change as a result.

Second, we did not test the effects of various socio-ecological factors, other than relational mobility, that researchers have proposed to affect the levels of culture-level individualism and collectivism, such as family structure (Greenfield, 2009; Yamawaki, 2012) local pathogen prevalence (Fincher et al., 2008), and regional differences in specific genes that regulate serotonergic neurotransmission and dopamine receptivity (Chiao and Blizinsky, 2009; Kitayama et al., 2014). Future research, perhaps using multi-ecology approach, is called for, in order to test whether these factors also have effects on the magnitude of association between self-esteem and happiness, and if yes whether the effect is independent from that of relational mobility.

Finally, still untested in Yuki et al.’s (2013) hypothesis is the exact process through which self-esteem maintains its strong association with happiness in high mobility environments (and less strongly in low mobility environments). Accordingly, it is predicted that such a pattern exists because, in the social domain, individuals with high social value are more likely to succeed (i.e., in making friends with someone with greater social value, etc.) than those with low social value and this is more visible in high mobility environments. Conversely, we would predict that social value would not count as much for social success in low mobility environments. Future research must test whether societies with varying levels of relational mobility really differ in this aspect of their social realities.

## Conflict of Interest Statement

The authors declare that the research was conducted in the absence of any commercial or financial relationships that could be construed as a potential conflict of interest.

## Footnotes

1 There were twelve participants who did not indicate the number of new acquaintances they had met during the past month.

2 Readers may wonder why the correlation between relational mobility, as assessed by the relational mobility scale, and the number of new friends was not so large. We argue that this is likely due to the difference in the nature of the two variables: with the former being an ecological variable (i.e., what the *society* is like), while the latter is an individual-level variable (i.e., what *I* am). The number of new friends, is thus affected not only by the relational mobility of the social ecology, but also by many other confounding factors, such as the person’s societal values (e.g., physical attractiveness, wealth, intelligence, etc.) and personality (e.g., extraversion). See Yuki and Schug (2012) for extensive discussion on this issue.

3 In our previous study, we found that the interaction between relational mobility and self-esteem effect on happiness mediated the interaction between participants’ culture (the U.S. vs. Japan) and self-esteem (Yuki et al., 2013, Study 1). However, in the current study, a conceptually parallel effect (mediation of the school year × self-esteem interaction by the relational mobility × self-esteem interaction) did not reach significance (*B* = -0.85, *t* = -1.45, *p* = 0.14). Although it is hard to explain why this was the case, we believe that the interpretation we offer is justifiable because (1) we used a quasi-experimental method with “minimal differences,” and (2) the manipulation check was successful. Moreover, there seems to be no other alternative theory that is more parsimonious than what we propose, which can account for all the findings regarding cross-cultural, cross-regional, and cross-situational differences in the association between self-esteem and happiness, such as the one indicated here.

## References

[B1] Baumeister R. F. Campbell J. D. Krueger J. I. Vohs K. D. (2003).Does high self-esteem cause better performance, interpersonal success, happiness, or healthier lifestyles? *Psychol. Sci. Public Interest* 4 1–44 10.1111/1529-1006.01431 26151640

[B2] Brown R. A. (2010). Perceptions of psychological adjustment, achievement outcomes, and self-Esteem in Japan and America. *J. Cross Cult. Psychol.* 41 51–61 10.1177/0022022109349507

[B3] Cheng H. Furnham A. (2003). Personality, self-esteem, and demographic predictions of happiness and depression. *Pers. Individ. Dif.* 34 921–942 10.1016/S0191-8869(02)00078-8

[B4] Chiao J. Y. Blizinsky K. D. (2009). Culture–gene coevolution of individualism–collectivism and the serotonin transporter gene. *Proc. R. Soc. B Biol. Sci.* 277 529–537 10.1098/rspb.2009.1650 PMC2842692 19864286

[B5] Deaton A. (2013). *The Great Escape: Health, Wealth, and the Origins of Inequality.* Princeton, NJ: Princeton University Press

[B6] Diener E. Biswas-Diener R. (2008). *Happiness: Unlocking the Mysteries of Psychological Wealth*. Trenton, NJ: John Wiley & Sons

[B7] Diener E. Diener M. (1995). Cross-cultural correlates of life satisfaction and self-esteem. *J. Pers. Soc. Psychol.* 68 653–663 10.1037/0022-3514.68.4.653 7738768

[B8] Diener E. Emmons R. A. Larsen R. J. Griffin S. (1985). The satisfaction with life scale. *J. Pers. Assess.* 49 71–75 10.1207/s15327752jpa4901_13 16367493

[B9] Falk C. F. Heine S. J. Yuki M. Takemura K. (2009). Why do Westerners self-enhance more than East Asians? *Eur. J. Pers.* 23 183–203 10.1002/per.715

[B10] Fincher C. L. Thornhill R. Murray D. R. Schaller M. (2008). Pathogen prevalence predicts human cross-cultural variability in individualism/collectivism. *Proc. Biol. Sci.* 275 1279–1285 10.1098/rspb.2008.0094 18302996 PMC2602680

[B11] Gelfand M. J. Raver J. L. Nishii L. Leslie L. M. Lun J. Lim B. C. (2011). Differences between tight and loose cultures: a 33-nation study. *Science* 332 1100–1104 10.1126/science.1197754 21617077

[B12] Greenfield P. M. (2009). Technology and informal education: what is taught, what is learned. *Science* 323 69–71 10.1126/science.1167190 19119220

[B13] Inglehart R. F. Foa R. Peterson C. Welzel C. (2008). Development, Freedom, and Rising Happiness A Global Perspective (1981-2007). *Perspect. Psychol. Sci.* 3 264–285 10.1111/j.1745-6924.2008.00078.x 26158947

[B14] James W. (1890/1983). *The Principle of Psychology.* Cambridge, MA: Harvard University Press (Original work published 1890)

[B15] Japanese Ministry of Health, Labor, and Welfare. (2006). *Annual Report on Health, Labor and Welfare 2006*. Tokyo

[B16] Japanese Ministry of Health, Labor, and Welfare Statistics and Information Department. (2014). *Vital Statistics in Japan. Trends up to 2012.* Tokyo

[B17] Japanese Ministry of Internal Affairs, and Communications. (2013). *Annual Report on the Labor Force Survey 2013.* Tokyo

[B18] Kahneman D. Krueger A. Schkade D. Schwarz N. Stone A. (2006). Would you be happier if you were richer? A focusing illusion. *Science* 312 1908–1910 10.1126/science.1129688 16809528

[B19] Kernis M. H. (ed.). (2006). *Self-Esteem Issues, and Answers: A Sourcebook of Current Perspectives*. New York: Psychology Press

[B20] Kitayama S. King A. Yoon C. Tompson S. Huff S. Liberzon I. (2014). The dopamine D4 receptor gene (DRD4) moderates cultural difference in independent versus interdependent social orientation. *Psychol. Sci.* 25 1169–1177 10.1177/0956797614528338 24747168

[B21] Kwan V. S. Y. Bond M. H. Singelis T. M. (1997). Pancultural explanations for life satisfaction: adding relationship harmony to self-esteem. *J. Pers. Soc. Psychol.* 73 1038–1051 10.1037/0022-3514.73.5.1038 9364759

[B22] Leary M. R. Baumeister R. F. (2000). “The nature and function of self-esteem: sociometer theory,” in *Advances in Experimental Social Psychology* Vol. 32 ed. Zanna M. P. (San Diego, CA: Academic Press) 1–62

[B23] Leary M. R. Tambor E. S. Terdal S. K. Downs D. L. (1995). Self-esteem as an Interpersonal monitor: the sociometer hypothesis. *J. Pers. Soc. Psychol.* 68 518–530 10.1037/0022-3514.68.3.518

[B24] Lyubomirsky S. (2008). *The How of Happiness: A Scientific Approach to Getting the Life You Want*. New York: Penguin Press

[B25] Markus H. R. Kitayama S. (1991). Culture and the self: implications for cognition, emotion, and motivation. *Psychol. Rev.* 98 224–253 10.1037/0033-295X.98.2.224

[B26] Markus H. R. Kitayama S. (2010). Cultures and selves: a cycle of mutual constitution. *Perspect. Psychol. Sci.* 5 420–430 10.1177/1745691610375557 26162188

[B27] Mecca A. M. Smelser N. J. Vasconcellos J. (eds). (1989). *The Social Importance of Self-Esteem.* Berkeley, CA: University of California Press

[B28] Mruk C. J. (2006). *Self Esteem Research, Theory, and Practice: Toward a Positive Psychology of Self Esteem*. New York: Springer

[B29] Nisbett R. E. Cohen D. (1996). *The Culture of Honor: The Psychology of Violence in the South*. Denver, CO: Westview Press

[B30] Oishi S. (2010). The psychology of residential mobility: implications for the self, social relationships, and well-being. *Perspect. Psychol. Sci.* 5 5–21 10.1177/1745691609356781 26162059

[B31] Oishi S. Graham J. (2010). Social ecology: lost and found in psychological science. *Perspect. Psychol. Sci.* 5 356–377 10.1177/1745691610374588 26162183

[B32] Oishi S. Schimmack U. (2010). Residential mobility, well-being, and mortality. *J. Pers. Soc. Psychol.* 98 980–994 10.1037/a0019389 20515253

[B33] Rosenberg M. (1965). *Society and the Adolescent Self-Image.* Princeton, NJ: Princeton University Press

[B34] Sato K. Yuki M. Norasakkunkit V. (in press). A socio-ecological approach to cross-cultural differences in the sensitivity to social rejection: the partially mediating role of relational mobility. *J. Cross Cult. Psychol.*

[B35] Schmitt D. P. Allik J. (2005). Simultaneous administration of the Rosenberg self-esteem scale in 53 nations: exploring the universal and culture-specific features of global self-esteem. *J. Pers. Soc. Psychol.* 89 623–642 10.1037/0022-3514.89.4.623 16287423

[B36] Schug J. Yuki M. Horikawa H. Takemura K. (2009). Similarity attraction and actually selecting similar others: how cross-societal differences in relational mobility affect interpersonal similarity in Japan and the United States. *Asian J. Soc. Psychol.* 12 95–103 10.1111/j.1467-839X.2009.01277.x

[B37] Schug J. Yuki M. Maddux W. (2010). Relational mobility explains between- and within-culture differences in self-disclosure toward close friends. *Psychol. Sci.* 21 1471–1478 10.1177/0956797610382786 20817913

[B38] Shirakawa M. (2011). “Globalization and population aging: challenges facing Japan,” in *Speech to the Board of Councillors of Nippon Keidanren* (Japan Business Federation), Governor of the Bank of Japan, Tokyo.

[B39] Sznycer D. Takemura K. Delton A. W. Sato K. Robertson T. Cosmides L. (2012). Cross-cultural differences and similarities in proneness to shame: an adaptationist and ecological approach. *Evol. Psychol.* 10 352 10.1177/147470491201000213 PMC3604996 22947644

[B40] Taylor S. E. Brown J. D. (1988). Illusion and well-being: a social-psychological perspective on mental health. *Psychol. Bull.* 103 193–210 10.1037/0033-2909.103.2.193 3283814

[B41] Uchida Y. Kitayama S. Mesquita B. Reyes J. A. S. Morling B. (2008). Is perceived emotional support beneficial? Well-being and health in independent and interdependent cultures. *Pers. Soc. Psychol. Bull.* 34 741–754 10.1177/0146167208315157 18359927

[B42] Uskul A. K. Kitayama S. Nisbett R. E. (2008). Ecocultural basis of cognition: Farmers and fishermen are more holistic than herders. *Proc. Natl. Acad. Sci. U.S.A.* 105 8552–8556 10.1073/pnas.0803874105 18552175 PMC2438425

[B43] Veenhoven R. (1999). Quality-of-life in individualistic society a comparison of 43 nations in the early 1990’s. *Soc. Indic. Res.* 48 157–186 10.1023/A:1006923418502

[B44] Yamagishi T. (2011). *Trust as Social Intelligence: The Evolutionary Game of Mind and Society*. New York: Springer

[B45] Yamagishi T. Hashimoto H. Li Y. Schug J. (2012). Stadtluft macht frei (city air brings freedom). *J. Cross Cult. Psychol.* 43 38–45 10.1177/0022022111415407

[B46] Yamawaki N. (2012). Within-culture variations of collectivism in Japan. *J. Cross Cult. Psychol.* 43 1191–1204 10.1177/0022022111428171

[B47] Yuki M. Sato K. Takemura K. Oishi S. (2013). Social ecology moderates the association between self-esteem and happiness. *J. Exp. Soc. Psychol.* 49 741–746 10.1016/j.jesp.2013.02.006

[B48] Yuki M. Schug J. (2012). “Relational mobility: a socio-ecological approach to personal relationships,” in *Relationship Science: Integrating Evolutionary, Neuroscience, and Sociocultural Approaches* eds Gillath O. Adams G. E. Kunkel A. D. (Washington, DC: American Psychological Association) 137–152

[B49] Yuki M. Schug J. R. Horikawa H. Takemura K. Sato K. Yokota K. (2007). *Development of a Scale to Measure Perceptions of Relational Mobility in Society.* CERSS Working Paper 75, Center for Experimental *Research in Social Sciences* Hokkaido University

